# Virtual Cancer Genomics: An Accessible and Effective Approach to Research Training for Undergraduates

**DOI:** 10.1007/s13187-025-02594-2

**Published:** 2025-02-24

**Authors:** Erica L. Gerace, Sarah Wojiski

**Affiliations:** Genomic Education, The Jackson Laboratory for Genomic Medicine, Farmington, CT USA

**Keywords:** Cancer research training, Undergraduate education, Virtual course, Inquiry project, Cancer genomics, Careers in cancer genomics

## Abstract

To diversify the cancer research workforce, it is necessary to broaden the accessibility of audience appropriate training programs. Cancer research training opportunities for undergraduates are often geographically bound and have limited enrollment capacities. To address this problem, the Genomic Education team at The Jackson Laboratory designed a free, 2-week, virtual short course in cancer genomics open to students across the USA. Each summer students learn foundational concepts in cancer genomics while gaining valuable exposure to a range of careers in the field. We developed recruitment strategies prioritizing students who are underrepresented in research and successfully scaled the course without compromising the student experience by creating tiered levels of engagement and flexibility through a mix of synchronous and interactive asynchronous activities. This approach accommodates students who work or are otherwise unable to participate in mentored research away from their home institution. In addition to 1-h live sessions designed for didactic content delivery and interaction with researchers and clinicians, the course offers participants in the highest tier of engagement collaboration in small groups on an inquiry project at the intersection of cancer genomics and patient care. Formal assessment of student attitudes indicates significant benefits and gains from participation in this course. Greater than ninety percent of surveyed students indicated the course was an effective way to learn about cancer genomics, had a positive effect on their interest in science, and provided clarification of their career path, which attests to the value of creating accessible training formats for undergraduates who wish to engage in biomedicine.

## Introduction

Diversity in the STEM workforce offers varied perspectives required to drive innovation, discovery, and development of new technology and research strategies [1]. Increasing diversity in training programs leads to a diversified cancer research and healthcare workforce which is essential to reducing existing cancer health disparities [2]. While the number of racial and ethnic minorities in cancer training programs has expanded over the last two decades, trainees who identify with these groups remain underrepresented compared to their overall representation in the US population and these groups, along with women, are underrepresented in the cancer research workforce [2, 3]. The American Association for Cancer Research (AACR) highlights that many training programs, including those for undergraduates, are geography-bound with inconsistent access across the country and, therefore, recommends enhancement of the trainee pipeline specifically suggesting the use of technology to improve access to and broaden dissemination of training [2].

Mentored research experiences are one way to shape researcher identity. Studies indicate that these experiences for undergraduates are particularly impactful and contribute to persistence of those traditionally underrepresented in STEM [4–6]. The Jackson Laboratory (JAX), a nonprofit biomedical research institute and National Cancer Institute (NCI)-designated basic cancer center, has a long-term commitment to providing opportunities for students and early career scientists to develop skills in genomics and to broaden participation in research. Since 1924, JAX Genomic Education (JAX GE) has invited undergraduate students from around the country to participate in 10-week mentored research experiences. Limited to 40 students, there are too few spots for every qualified applicant.

Answering AACR’s call to use technology to enhance the training pipeline, JAX GE designed an accessible 2-week *Cancer Genomics* virtual short course featuring a mix of synchronous sessions and asynchronous activities. Enrolled students learn the biology of cancer cells, the bioinformatic approaches used to study cancer genomes, and explore the intersection of cancer genomics research with clinical care. They discover how genetic alterations in cancer genomes inform cancer treatment and learn the ethical framework that guides patient care and human subjects research. This course combines best practices for virtual pedagogy and active learning strategies to promote self-efficacy associated with persistence in science and research fields [7, 8]. *Cancer Genomics* includes large and small group interactivity fostering engagement as well as a web-based inquiry project, a viable alternative for large enrollment courses leading to positive learning gains comparable to those realized in laboratory-based projects [9, 10]. Synchronous sessions featuring invited speakers who are researchers, bioinformaticians, and clinical professionals are an effective means to connect students to possible career paths within a field [11, 12].

Here, we describe the design of *Cancer Genomics* and detail how through prioritized recruitment we successfully scaled this virtual course and increased the enrollment of students who attend minority-serving institutions (MSIs). We also summarize evaluation results that affirm the course clarifies career direction and is an effective way to improve understanding of science and research. The course outlined here can serve as an example of how a virtual platform can provide an accessible training opportunity reaching diverse groups of undergraduates.

## *Cancer Genomics*, a Virtual Short Course

### Course Design

The goals of the *Cancer Genomics* course are to (1) teach foundational concepts in cancer genomics, (2) introduce students to approaches used to study cancer genomes, and (3) provide exposure to different careers in the field of cancer genomics, using a virtual platform that is flexible and accessible to undergraduates across the United States. Over four iterations from 2021 to 2024, the course has evolved, incorporating best practices in teaching that foster student engagement while accommodating large enrollments without compromising the student experience. The pilot instance with 26 students in 2021 established the course structure with two components: (1) synchronous sessions including didactic lessons, guest speaker presentations, and interactive group work and (2) asynchronous engagement using a learning management system for formative assessment of understanding and collaborative discussion boards prompting reflection. All synchronous sessions take place on Zoom, and asynchronous work is completed at each student’s pace. Members of the JAX GE team who are trained cancer researchers deliver didactic content lessons, and invited speakers present their cancer research projects or describe the impact of genomics in their clinical careers. Speakers demonstrate the application of cancer genomics concepts in research and clinical care, providing valuable career exposure for students considering their career paths [11, 12].

The course includes an inquiry project where students assume the role of a clinical oncologist and analyze data from a fictitious patient who received genomic tumor testing to identify potentially pathogenic gene variants. Groups of five to six students evaluate the consequence of genetic variation on biological function, classify variant clinical significance, and identify actionable therapies. The project is scaffolded through checkpoint assignments that guide students through cancer genomics databases, such as cBioPortal and OncoKB. Ultimately, each group creates a finalized genomic tumor testing patient report detailing data collected about gene variants identified in their fictious patient’s tumor and associated actionable therapies. They also assemble a short presentation detailing a treatment strategy for their patient that is delivered on the last day of the course.

### Tiers of Engagement

**Fig. 1:**
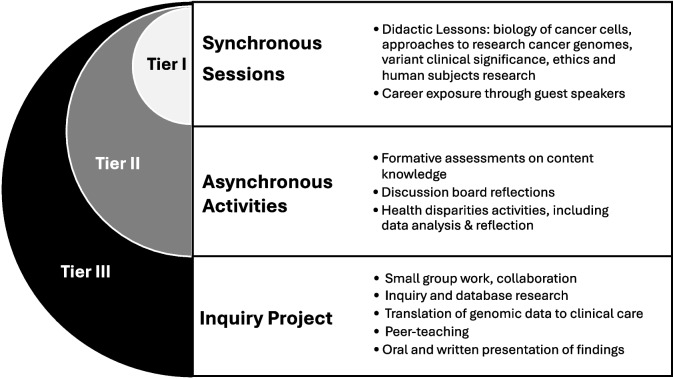
Tiers of engagement offer flexibility for student enrollment and time commitment. Three tiers of engagement build on each other and Tier III participants engage with all three course elements

Over several course iterations, we observed that some participants who were unable to complete every course element stopped attending altogether. In response, in 2024, we created concurrent tiers of engagement that build upon each other, providing flexibility within the course structure and further enhancing accessibility (Fig. 1). Tier I participants attend large group synchronous sessions, totaling 6 h over 2 weeks. Participants in Tier II attend the same large group synchronous sessions, and complete asynchronous group discussions, reflections, and assignments on the learning management system, requiring up to four additional hours outside of class. Tier III students attend all synchronous sessions, complete asynchronous activities, and join small groups to conduct inquiry projects for an additional 10 h over the 2 weeks (20 total hours). Students who complete Tier III earn a digital badge microcredential issued through Credly®, while those who complete Tier II instead earn a certificate of completion. In 2024, of 229 course participants, 108 earned a digital badge and 74 earned a certificate (Table 1). This strategy accommodates students who may have other obligations and cannot dedicate significant time outside of class to complete asynchronous work. While Tier I students will not benefit from the inquiry project or deeper engagement, they receive content presented in didactic lessons and are exposed to possible career paths by attending guest speaker presentations. With flexibility to shift tiers at the start of the class, students can assess the workload and choose their engagement accordingly.

**Table 1:** Course participant demographics and academic status over four iterations from 2021 to 2024

	**2021**	**2022**	**2023**	**2024**	**Total**
Total	26	17	102	229	374
**Demographics**					
Racial/ethnic minority	5 (19%)	7 (41%)	54 (53%)	134 (59%)	200 (53%)
Disadvantaged background	n.d	4 (24%)	n.d	94 (41%)	n.d
Women	18 (69%)	14 (82%)	77 (75%)	171 (72%)	280 (75%)
First generation	n.d	n.d	50 (49%)	104 (45%)	n.d
**School**					
No. of colleges and universities represented	18	11	38	120	164
Attending Minority serving institution^**^	4 (15%)	8 (47%)	88 (86%)	134 (59%)	234 (63%)
AANAPISI^*^	2	6	46	37	91
ANNH^†^	0	0	1	0	1
HBCU^‡^	0	1	6	18	25
HSI^§^	3	7	63	92	165
PBI^¶^	0	0	7	1	8
TCU^#^	0	0	0	2	2
**Major**					
Life Sciences	21 (81%)	15 (88%)	94 (92%)	177 (77%)	307 (82%)
Health Sciences	3 (12%)	0	2 (2%)	16 (7%)	21 (6%)
Computational Biology/Bioinformatics	0	0	0	6 (3%)	6 (2%)
Engineering	1 (4%)	0	1 (1%)	10 (4%)	12 (3%)
STEM, other	0	0	2 (2%)	8 (3%)	10 (3%)
Undeclared/other	1 (4%)	2 (12%)	3 (3%)	12 (5%)	18 (4%)
Undergraduate year					
2nd Year	7 (27%)	5 (29%)	42 (41%)	42 (18%)	96 (26%)
3rd Year	7 (27%)	3 (18%)	26 (25%)	64 (28%)	100 (27%)
4th Year	10 (38%)	8 (47%)	30 (29%)	97 (42%)	145 (39%)
Other/N.R	2 (8%)	1 (6%)	4 (4%)	26 (11%)	33 (9%)
**Engagement tiers**					
Completed Tier I	Not offered	Not offered	Not offered	47	
Completed Tier II				74	
Completed Tier III				108	

Demographic data including racial or ethnic identity, gender identity, reported disadvantaged background as previously defined by *NIH* *NOT-OD-20–031*, and first-generation status were collected and are shown as a number and percentage for each year and for the lifetime of the course (total).

^*^Asian American and Native American Pacific Islander-Serving Institutions

^†^Alaska Native-Serving and Native Hawaiian-Serving Institutions

^‡^Historically Black Colleges and Universities

^§^Hispanic-Serving Institutions

^¶^Predominantly Black Institutions

^#^Tribal Colleges and Universities

^**^MSI status is federally determined and some schools have more than one status

### Prioritized Recruitment

In four instances, 374 undergraduates from 164 colleges and universities in 37 states, the District of Columbia, and Puerto Rico completed the course. The institutions varied from large public universities to small private liberal arts schools to 2-year colleges. Eighty-two percent of course participants reported majoring in life sciences, although there are increasing numbers of computational biology, bioinformatics, and engineering majors (Table 1). We recommend that students complete 1 year of college before enrolling to increase the likelihood of familiarity with concepts covered in introductory biology; therefore, most students were entering their second, third, or fourth years (Table 1). Notably, we consistently enrolled a large portion of female-identifying students, 75% overall (Table 1).

While the course is open to any undergraduate from any college or university, we focused recruitment on minority-serving institutions (MSIs) to increase access to students from groups traditionally underrepresented in cancer research. We curated a list of life science faculty and advisors at MSIs and emailed this list information about the course to share with their students. In 2023, the first year of prioritized recruitment, 86% of 102 students attended an MSI, 73% identified with minority racial and ethnic groups, and 45% reported being a first-generation college student (Table 1). In 2024, we increased enrollment to 229 with 59% attending an MSI, 59% reporting identities with racial and ethnic minority groups, 41% from disadvantaged educational backgrounds, and 31% in more than one category as previously defined by the *Notice of NIH’s Interest in Diversity NOT-OD-20–031* (Table 1, data not shown).

### Course Evaluation

**Fig. 2:**
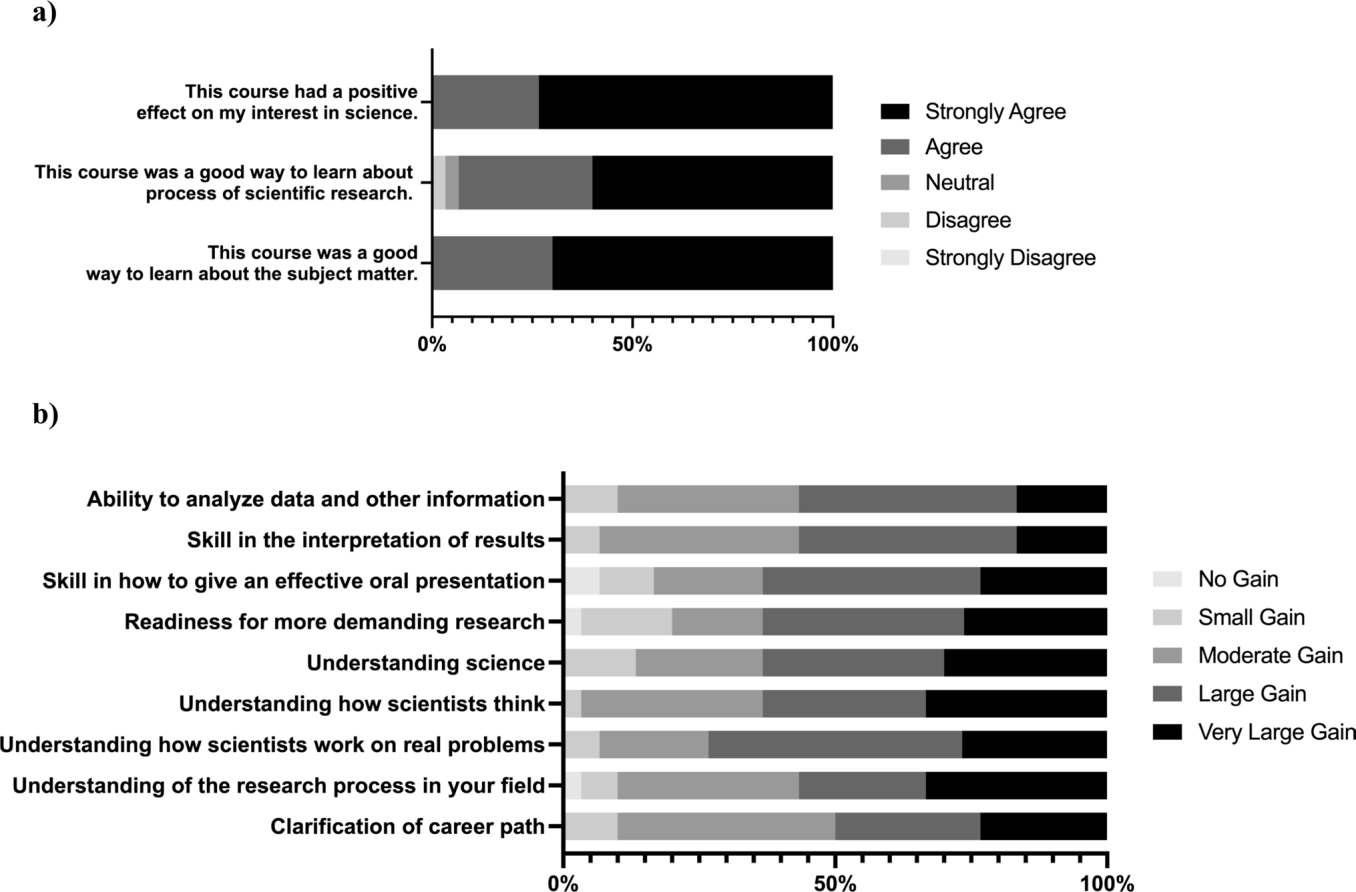
Course participants report positive benefits, gains in research skills, and clarification of career path. Percentages were calculated from total post-survey responses (*n* = 30) to Likert scale items on the CURE post-survey instrument to evaluate the overall course (**a**) and course benefits (**b**) [13]

To formally assess if this course (1) is an effective way of learning about cancer genomics and about conducting research in this field and (2) has a positive impact on interest in science and pursuing career paths in research, we identified the Classroom Undergraduate Research Experience (CURE) survey as a validated instrument to evaluate “research like” undergraduate courses [13]. We invited Tier III students in the 2024 course to take a pre-survey a week before course commencement and a post-survey within a week of course completion to assess their perceptions and attitudes. We focused the study on Tier III students as they engaged with each course element and completed the inquiry project. Considering the course duration of 2 weeks and the fact that only 30 students completed both pre- and post-course surveys (see Limitations), as expected, it was difficult to discern changes. Using paired *t*-tests from pre- and post-survey data (*n* = 30), we did not uncover significant changes regarding student opinions of science or increases in perceived ability to perform course elements (data not shown). However, data collected on the post-survey, including the course evaluation (Fig. 2a) and measurement of benefits gained (Fig. 2b), demonstrated positive outcomes.

The overall course evaluation data, shown in Fig. 2a, indicate students strongly agreed or agreed that the course was a good way learn about the subject matter (100%) and about the process of scientific research (93%). In addition, 100% of students strongly agreed or agreed that the course had a positive effect on their interest in science (Fig. 2a).

Students reported gains in competencies associated with research including ability to analyze data and skill in the interpretation of results with 90% indicating at least a moderate gain for each (Fig. 2b). Students also reported skill gains in how to give an effective oral presentation, which is important for communication as a researcher or in biomedical careers (Fig. 2b). Eighty-one percent of students reported at least a moderate gain in readiness for more demanding research (Fig. 2b). Most students had at least moderate gains in their understanding of science (86%), the research process in their field (89%), how scientists think (93%), and how scientists work on real problems (94%) (Fig. 2b). Each of these gains is consequential as a majority reported they are heading towards research-focused graduate programs. Sixty-six percent of post-survey responders indicated their goal is to attend graduate school for either a Ph.D. in a biology-related field (43%) or Masters in a STEM field (23%) (data not shown). Importantly, 90% of students reported at least a moderate gain in clarification of career path with 50% naming a large or very large gain (Fig. 2b).

### Limitations

One limitation of this study is the evaluation of participant outcomes. The course is ungraded and only 2 weeks long, which we suspect impacted our capacity to detect changes in attitudes about science and research between pre- and post-course surveys. We also had to rely on digital communication to distribute surveys, which contributed to a low response rate particularly on the post-course survey that only 30 of 108 students completed. However, the demographics of the post-survey responders do reflect that of the entire group who completed the highest engagement course tier.

Another challenge is student enrollment and retention. The Jackson Laboratory is a not a degree-granting institution, and we do not provide university or college credit. While we do not charge students to take the course and offer a microcredential to students who qualify upon completion, we notice significant attrition from the time of registration. We also observed shifts in demographics when comparing the group that registered for and the group that ultimately completed Tier III, the most demanding level of the course. We do not know the specific motivations behind these shifts to a lower tier of engagement in the first week of the course. However, collecting data from subsequent iterations could allow us to pinpoint the factors influencing tier changes, and we could modify the course to make it more accessible to the most vulnerable students or those that could benefit most from deeper engagement.

## Discussion

To build the diverse cancer research workforce needed to address existing cancer disparities, it is vital to create open access training opportunities at all levels, especially for students in underserved or under resourced areas [2]. Undergraduates are at a particularly impressionable stage of their training when small research experiences can significantly influence persistence in their chosen field of study and impact their ultimate career choice [5, 7]. Accessible training programs that foster a sense of belonging and provide opportunity to build self-efficacy in skills required for research increase the likelihood for persistence in research and STEM fields [6, 14]. This is particularly important for those who identify with groups traditionally underrepresented or who come from disadvantaged backgrounds [15].

*Cancer Genomics* provides opportunity for students to build self-efficacy in skills related to success in research careers. Course participants who completed the web-based inquiry project reported gains in ability to perform data analysis and results interpretation as well as gains in understanding of science and the research process in the field (Fig. 2b). This data corroborates the finding that web-based research can lead to similar gains as those identified with laboratory-based projects [10], yet they require significantly fewer resources and reach students in their home locations. Accessible research projects are particularly important for students who may have summer obligations, cannot take part in a lengthy mentored research program, or do not have the background or support to vie for a spot in such a program [16]. *Cancer Genomics* adds to growing evidence that virtual experiences can be as effective as in-person, further supporting the importance of creating training with flexible engagement particularly for those from underserved or disadvantaged backgrounds [17, 18].

Fostering sense of belonging involves cultivating an inclusive community that provides support for students and their learning, which ultimately leads to forming science identity and motivation to pursue a research career [19, 20]. Our post-survey responders indicate that one benefit of *Cancer Genomics* was joining a learning community with 83% reporting at least a moderate gain and 66% reporting a large or very large gain (data not shown). We attribute this gain to the design of this virtual course that connects participants from many different backgrounds and locations across the country. To do this, we capitalized on features of the technology to incorporate interactivity into each component of the course to encourage active participation. In large group sessions, we invited participant questions and integrated chats, polls, and word clouds. In each small group section, we incorporated ice breakers to help students get to know each other. Asynchronous activities included discussion board posts for clarification, interacting with peers, and receiving feedback from instructors. One key factor contributing to the establishment of an inclusive community is the approachability of faculty, and 100% of post-survey responders agreed or strongly agreed they were able to ask questions and get helpful responses in this class (data not shown) [20].

Here, we show that The Jackson Laboratory’s short course *Cancer Genomics* has met the goal to provide research training and career exposure to undergraduates that is accessible to students across the country. Enrolling mostly students from MSIs including those serving Hispanic, Black, and Native American populations, *Cancer Genomics* is meaningful training opportunity for students who hopefully will go on to diversify the cancer workforce.

## Data Availability

De-identified data will be provided upon request.
