# Correction: Virtual Cancer Genomics: An Accessible and Effective Approach to Research Training for Undergraduates

**DOI:** 10.1007/s13187-025-02612-3

**Published:** 2025-03-25

**Authors:** Erica L. Gerace, Sarah Wojiski

**Affiliations:** Genomic Education, The Jackson Laboratory for Genomic Medicine, Farmington, CT USA

**Correction: Journal of Cancer Education**

10.1007/s13187-025-02594-2

The original version of this article contained mistakes.

Figures 1 and 2 in the original version of this article have been replaced.

The original article has been updated.

